# Psychological hallmarks of endometriosis with emphasis on sexual dysfunction, stress, anxiety and depression

**DOI:** 10.1192/j.eurpsy.2023.1345

**Published:** 2023-07-19

**Authors:** R. Sajdlova, L. Fiala

**Affiliations:** Sexology department, Faculty of medicine in Pilsen, Charles University, Pilsen, Czech Republic

## Abstract

**Introduction:**

More than 50% of women with endometriosis report that they suffer from sexual dysfunctions, the most significant of which is pain, which can subsequently be associated with stress, anxiety, depression. The aim of this study was to evaluate the relationship between sexual function, stress, anxiety and depression together with the values of stress hormones such as cortisol and prolactin in women with endometriosis.

**Objectives:**

Endometriosis can occur in up to 15% of women. A characteristic feature is the presence of tissue resembling the endometrium outside the uterine cavity. Endometriosis is an estrogen-dependent disease that is associated with fertility disorders (incidence up to 40%) and sexual dysfunction (up to 50% of patients). Endocrine and immune changes may be associated with chronic stress, anxiety and even depression.

**Methods:**

A total of 92 patients with endometriosis were included in the study. Clinical examinations were focused on biochemical analysis of cortisol and prolactin. At the same time, sexual function, symptoms of stress, anxiety and depression were psychometrically evaluated in these patients.

**Results:**

In the mutual statistical assessment, positive correlations were found between the results of the Beck scale questionnaire for assessing the severity of depression (BDI-II) and PRL (R = 0.39), then confirmed by Mann-Whitney test (z-score is 5.98019, p value is <0.00001, result is significant at p <0.05). Furthermore, the correlation between BDI-II and HAM-A (R = 0.33), confirmed by the Mann-Whitney test (z-score is -8.55827, p value is <0.00001, the result is significant at p <0.05) (Fig.). Positive correlations were found between TSC-40 and FSDS-R (R = 0.30), confirmed by Mann-Whitney test (z-score is 3.89503, the value of p is 0., 0001, the result is significant at p <0.05). We also found a high correlation between PRL and HAM-A (R = 0.86). Cortisol levels did not show any positive correlation.

**Conclusions:**

Sexual dysfunction usually accompanies up to 50% of patients with endometriosis. This in turn affects the mental health and well-being of not only the patients, but also their sexual partners. The results of this study are consistent with the hypothesis of possible relationships between stress, anxiety, depression and neuroendocrine markers in patients with endometriosis.

Women diagnosed with the symptoms of endometriosis should also be examined for psychosocial and psychiatric disorders at the same time. In this regard, it is important not to underestimate the psychological assessment of those patients who are at risk of developing symptoms of anxiety and depression and to provide them with appropriate psychological support.

**Disclosure of Interest:**

None Declared

